# An Automated Line-of-Therapy Algorithm for Adults With Metastatic Non–Small Cell Lung Cancer: Validation Study Using Blinded Manual Chart Review

**DOI:** 10.2196/29017

**Published:** 2021-10-12

**Authors:** Weilin Meng, Kelly M Mosesso, Kathleen A Lane, Anna R Roberts, Ashley Griffith, Wanmei Ou, Paul R Dexter

**Affiliations:** 1 Center for Observational and Real-World Evidence Merck & Co, Inc Kenilworth, NJ United States; 2 Department of Biostatistics and Health Data Science Indiana University School of Medicine Indianapolis, IN United States; 3 Regenstrief Institute, Inc Indianapolis, IN United States; 4 Eskenazi Health Indianapolis, IN United States; 5 Department of Medicine Indiana University School of Medicine Indianapolis, IN United States

**Keywords:** automated algorithm, line of therapy, longitudinal changes, manual chart review, non–small cell lung cancer, systemic anticancer therapy

## Abstract

**Background:**

Extraction of line-of-therapy (LOT) information from electronic health record and claims data is essential for determining longitudinal changes in systemic anticancer therapy in real-world clinical settings.

**Objective:**

The aim of this retrospective cohort analysis is to validate and refine our previously described open-source LOT algorithm by comparing the output of the algorithm with results obtained through blinded manual chart review.

**Methods:**

We used structured electronic health record data and clinical documents to identify 500 adult patients treated for metastatic non–small cell lung cancer with systemic anticancer therapy from 2011 to mid-2018; we assigned patients to training (n=350) and test (n=150) cohorts, randomly divided proportional to the overall ratio of simple:complex cases (n=254:246). Simple cases were patients who received one LOT and no maintenance therapy; complex cases were patients who received more than one LOT and/or maintenance therapy. Algorithmic changes were performed using the training cohort data, after which the refined algorithm was evaluated against the test cohort.

**Results:**

For simple cases, 16 instances of discordance between the LOT algorithm and chart review prerefinement were reduced to 8 instances postrefinement; in the test cohort, there was no discordance between algorithm and chart review. For complex cases, algorithm refinement reduced the discordance from 68 to 62 instances, with 37 instances in the test cohort. The percentage agreement between LOT algorithm output and chart review for patients who received one LOT was 89% prerefinement, 93% postrefinement, and 93% for the test cohort, whereas the likelihood of precise matching between algorithm output and chart review decreased with an increasing number of unique regimens. Several areas of discordance that arose from differing definitions of LOTs and maintenance therapy could not be objectively resolved because of a lack of precise definitions in the medical literature.

**Conclusions:**

Our findings identify common sources of discordance between the LOT algorithm and clinician documentation, providing the possibility of targeted algorithm refinement.

## Introduction

Lung cancer is the most common cause of cancer-related deaths worldwide [1], accounting for almost 2 million deaths annually [2,3]. Non–small cell lung cancer (NSCLC) represents approximately 85% of all lung cancer cases [4]. Treatment for advanced NSCLC is increasingly based on molecular patterns, including therapies that target mutations such as *EGFR* and *ALK* genomic aberrations, as well as inhibitors of the programmed death 1 (PD-1) pathway, particularly for patients whose tumors have high levels of PD-ligand 1 expression [5]. Although survival for patients with advanced disease has improved, the need for continued therapeutic advances and research remains acute [4].

Cancer therapy is commonly classified into lines of therapy (LOTs), each comprising one or more cycles of a single agent or a combination systemic anticancer therapy (SACT) [6-8]. Extraction of LOT data from real-world transactional claims and electronic health records (EHRs) is essential for determining longitudinal SACT changes in real-world clinical care settings, but it is challenging because LOT information is often not clearly marked in structured data sets and therefore must be interpreted through clinical notes [6,9]. Researchers and clinicians use LOT information gathered retrospectively to determine the effectiveness of SACT regimens, identify trends in clinical practice patterns, identify eligible candidates for cancer trials, and conduct quality assurance to help ensure that patients receive optimal SACT [6,10,11]. Manual determination of LOT information for large numbers of patients is time consuming and often not feasible, prompting our own and others’ searches for automated LOT algorithmic methods [6,12-15].

The objective of this study is to validate and refine our previously described open-source LOT algorithm [6] by comparing the LOT algorithm output with results obtained through independent blinded manual chart review.

## Methods

### Study Design, Patient Selection, and Data Extraction

After receiving approval from the Indiana University Institutional Review Board, we conducted a retrospective cohort analysis using structured EHR data and clinical documents from the Indiana Network for Patient Care, one of the largest and oldest health information exchanges in the United States [16-18]. The Indiana Network for Patient Care holds more than 13 billion data elements from more than 100 separate health care entities, including more than 130 million clinical documents providing data on nearly 15 million patients.

To validate the LOT algorithm, we identified adult patients treated for metastatic NSCLC with SACT and excluded patients who had received any SACT commonly used for small cell lung cancer, as described in Multimedia Appendix 1. To select the study cohort, we used the first iteration of the LOT algorithm to identify all the complex cases in the initial eligible population because we wanted to oversample patients with complex treatment sequences to train the algorithm. *Complex cases* were defined as patients who had either a maintenance therapy or more than one LOT, whereas *simple cases* were defined as those with only one LOT and no maintenance therapy. The complex cases were automatically selected for confirmation by chart review, and then simple cases were randomly chosen to complete the sample of 500 patients. The final determination of simple versus complex cases was thus made via chart review conducted by a physician (PRD).

Next, we extracted structured data commonly found in claims data and required by the LOT algorithm, including patient identifiers, SACT medications, and associated dates. For SACT medications, we filtered the SACT drug list to those used to treat metastatic NSCLC. The index date of the first-line (L1) treatment in this study corresponded to the date of initial SACT on or after recorded evidence of metastatic disease. For chart review purposes, we extracted all available clinical notes after the metastatic diagnosis date. In preparation for manual chart review, these clinical notes were loaded into nDepth, the Regenstrief natural language processing platform. This platform provides an efficient means of reviewing documents and capturing related information on a per-patient basis.

We then created a CSV file with patient identifier number, administration start date, administration end date (for oral drugs), and generic drug name as the column fields. This format is the minimum information required for the LOT algorithm input. Finally, we divided patients into a training cohort of 70% (350/500) patients and a test cohort of 30% (150/500) patients, using stratified sampling to keep the ratio of simple:complex cases the same in both cohorts. All algorithmic changes were performed using the training cohort data, after which the final version of the algorithm was evaluated against the test cohort.

### LOT Algorithm

Investigators at Merck Sharp & Dohme Corp have internally developed automated business rules to identify LOT numbers, treatment regimens, and maintenance treatment for patients with cancer [6]. These rules consist of tumor-agnostic algorithmic processes that extract LOT information from claims databases or EHRs. Implemented as R (The R Foundation for Statistical Computing) and Python (Python Software Foundation) code routines, the LOT algorithm uses a modular design to facilitate plug-and-play tumor-specific customization. The LOT algorithm along with tumor-specific customizations is available as open-source software through GitHub [19].

**Figure 1:**
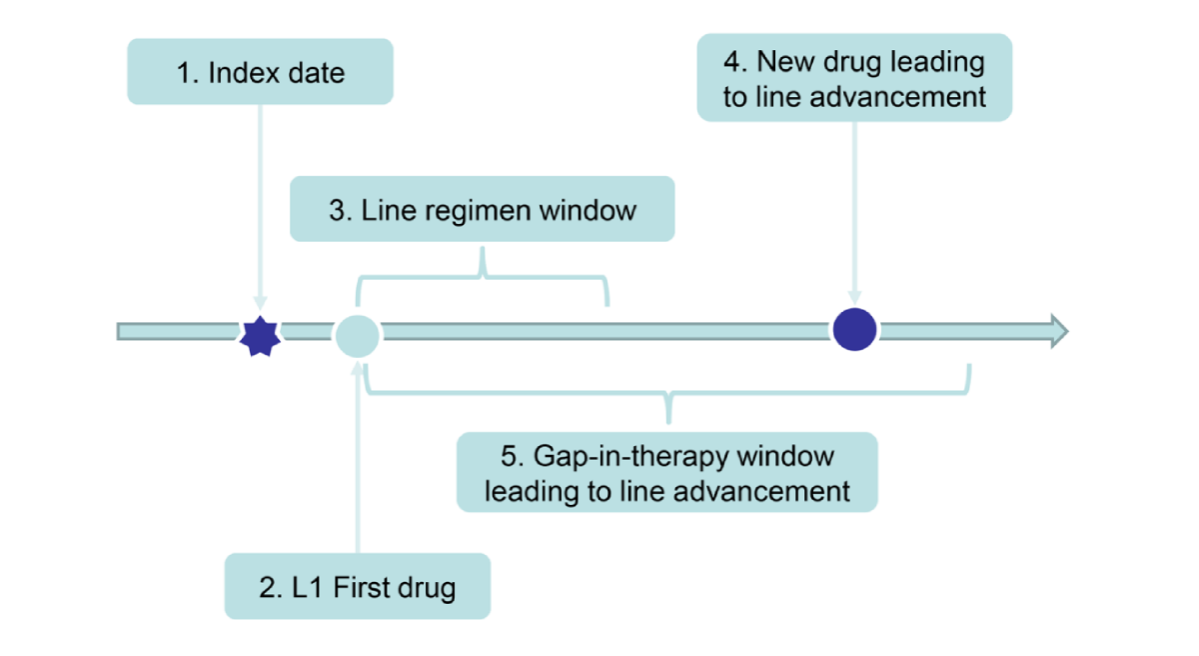
Schematic depicting the five basic modules of a line-of-therapy (LOT) algorithm. L1: first-line therapy. Reprinted with permission from Meng et al [6].

An overview of the LOT algorithm can be roughly understood by breaking it down into the five basic modules depicted in Figure 1: (1) the index date is defined as the date of the metastatic NSCLC diagnosis; (2) the L1 first drug is defined as the first SACT drug claim recorded at any time on or after the index date; (3) the line regimen window is the time starting with the L1 first drug and extending forward in time, typically for 28 days, to capture any other drugs administered in combination with the first drug, and the resulting set of drugs defines that LOT treatment regimen; (4) line advancement occurs if a new drug not belonging to the treatment regimen is introduced; and (5) line advancement also occurs if a drug is administered after a long gap in therapy.

Within these modules, several parameters are available in the code that allow the adjustment and introduction of special cases and exceptions for the rules. Common adjustments relate to the detection of maintenance therapy, checking for drug switches early in a LOT, and adding exceptions to line advancement for gaps in therapy or when certain drug classes are added or substituted in a treatment regimen.

### Blinded Manual Chart Review and Initial (Prerefinement) Validation of NSCLC Output

Blinded to results generated from the LOT algorithm, a physician (PRD) used the nDepth chart review functionality to review clinical notes for patients with metastatic NSCLC. The reviewer also had access to a spreadsheet that included the individual SACT medication names and dates of administration for each patient. The majority of detailed SACT LOT and maintenance therapy descriptions came from outpatient oncology notes. The reviewer extracted the following clinical information for each patient: (1) the sequence of SACT LOT and (2) maintenance therapy. He then formatted this information in a spreadsheet format identical to the LOT algorithm output to facilitate automated comparison.

For the initial (prerefinement) validation, we customized the NSCLC LOT algorithm parameters using the previously published criteria [6]. We then evaluated the output of the NSCLC LOT algorithm and compared it with the findings from chart review.

### NSCLC LOT Algorithm Refinement and Subsequent (Postrefinement) Validation

After completion of the blinded, automated initial comparison between algorithm output and chart review for the patients in the training cohort (n=350), we identified issues accounting for any discordance between algorithm output and chart review. To evaluate the areas of discordance, we separated the cases into simple and complex categories. For each issue, we then refined the LOT algorithm using close review of the initial comparison results, iterative rerunning of the refined LOT algorithm against the original chart review results, discussion with internal experts, and targeted medical literature review. Researchers from Merck Sharp & Dohme, Indiana University, and Regenstrief reviewed the deidentified raw SACT data and arbitrated the differences between algorithm output and chart review through a series of meetings.

### Statistical Analysis

Descriptive statistics were calculated for demographic and LOT characteristics, including means, SDs, ranges for continuous variables, and counts and percentages for categorical variables. One-way analysis of variance (ANOVA) and the Fisher exact test, as appropriate, were used to compare demographic characteristics and LOT counts between the training and test cohorts.

Intraclass correlation coefficients (ICCs) and the corresponding 95% CIs for the number of LOTs for each case based on the LOT algorithm and chart review were calculated. Percentage agreement and 95% CIs were calculated to compare the results from the LOT algorithm with the chart review. Agreement was defined as an exact match between the LOT algorithm output and physician chart review in terms of LOT number, regimen name, and maintenance therapy classification. Each LOT comprised the treatment as well as any subsequent maintenance therapy regimen.

## Results

### Cohort Selection

We identified 11,223 patients with at least one diagnosis code for lung cancer during the study period. Of these, 1461 patients had metastatic lung cancer as defined by diagnosis codes, metastatic criteria, and receipt of SACT 14 days before or any time after the index date. Of these 1461 patients, 897 patients also had NSCLC mentioned in unstructured patient notes.

**Figure 2:**
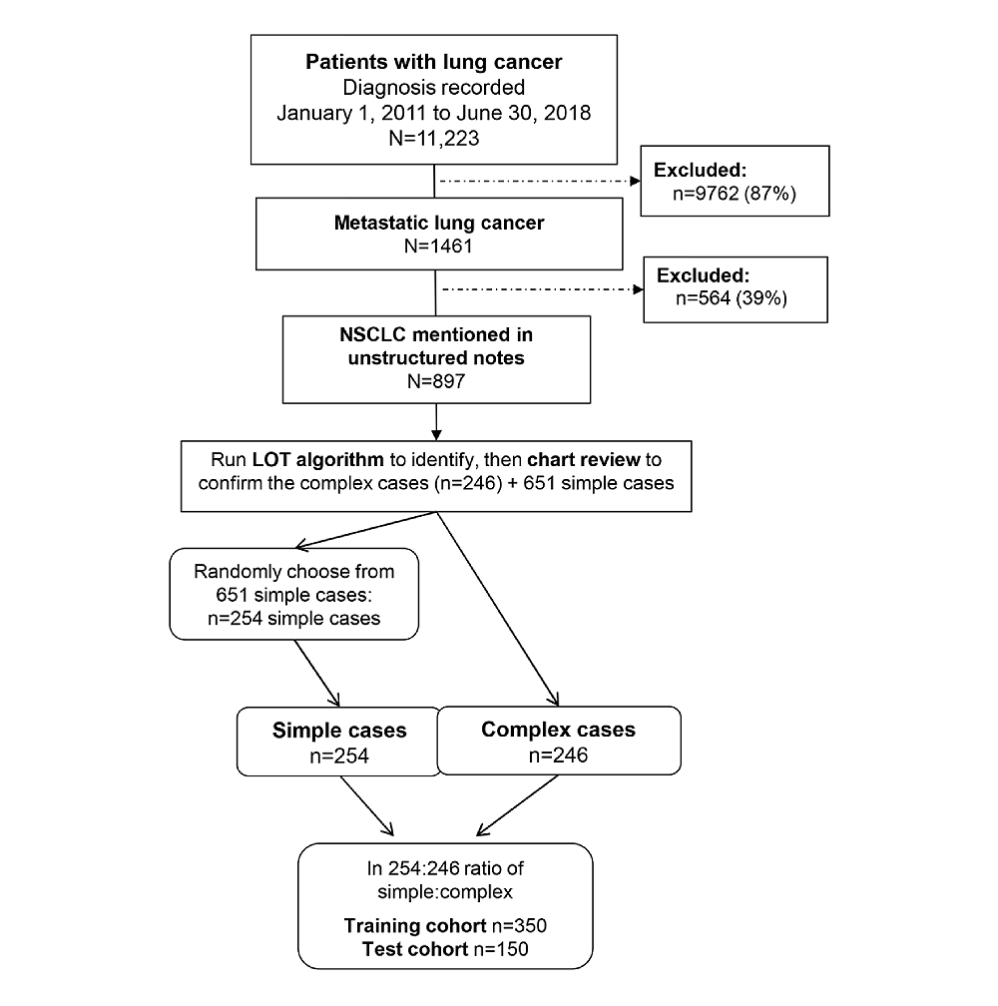
Selection of 500 patients whose deidentified charts were included in the study. LOT: line of therapy; NSCLC: non–small cell lung cancer.

To construct our final sample, the first iteration of the LOT algorithm was run on the 897 eligible patients. All complex cases who, according to the algorithm output, received more than one LOT and/or maintenance therapy were automatically selected for chart review. The chart review identified 246 patients as complex cases, and then 254 patients who had only a single LOT and never received maintenance therapy (simple cases) were randomly chosen to complete the sample of 500 patients. The 500 cases were then split into training and test cohorts with the same ratios of simple:complex cases (Figure 2).

No significant differences in patient characteristics were found between the training and test cohorts (Table 1).

**Table 1:** Patient demographic characteristics.

Demographics		All patients (N=500)	Training cohort (n=350)	Test cohort (n=150)	*P* value	
Female, n (%)		220 (44.0)	153 (43.7)	67 (44.7)	.85^a^	
**Age (years)**						.16^b^
	Mean (SD)	64.3 (10.7)	64.8 (10.7)	63.3 (10.5)		
	Range	25-91	34-91	25-90		
**Race,^c^ n (%)**						.95^a^
	White	442 (89.1)	308 (88.8)	134 (89.9)		
	Black	50 (10.1)	36 (10.4)	14 (9.4)		
	Asian	4 (0.8)	3 (0.9)	1 (0.7)		
Hispanic, Latino, or other ethnicity		2 (0.4)	1 (0.3)	1 (0.7)	.49^a^	

^a^Fisher exact test comparing training and test cohorts.

^b^Linear model analysis of variance comparing training and test cohorts.

^c^No information on race was available for 4 patients.

### Blinded Manual Chart Review Findings

The distributions of LOT counts were similar between the training and test cohorts, and simple and complex cases each represented approximately half of the cases (Table 2).

A total of 55.1% (193/350) patients in the training cohort received one LOT. An additional 29.4% (103/350) had two LOTs, and 10.3% (36/350) had three LOTs. Most patients had three or fewer LOTs during their treatment history, and 14.6% (51/350) patients received maintenance therapy (Table 2).

**Table 2:** Blinded manual chart review findings for lines of therapy.

Group			All patients (N=500)		Training cohort (n=350)		Test cohort (n=150)		*P* value	
**Case classification, n (%)^a^**										N/A^b^
	Simple cases	254 (50.8)		178 (50.9)		76 (50.7)				
	Complex cases	246 (49.2)		172 (49.1)		74 (49.3)				
**LOT,^c^ n (%)**										.09^d^
	1	280 (56.0)		193 (55.1)		87 (58.0)				
	2	144 (28.8)		103 (29.4)		41 (27.3)				
	3	51 (10.2)		36 (10.3)		15 (10.0)				
	4	20 (4.0)		17 (4.9)		3 (2.0)				
	5	3 (0.6)		0 (0.0)		3 (2.0)				
	6	2 (0.4)		1 (0.3)		1 (0.7)				
Maintenance therapy, n (%)			74 (14.8)		51 (14.6)		23 (15.3)		.89^d^	

^a^Blinded manual chart review was used to identify simple cases as patients who received one line of therapy (LOT) and no maintenance therapy, and complex cases as patients who received more than one LOT and/or maintenance therapy.

^b^N/A: not applicable.

^c^LOT: line of therapy.

^d^Fisher exact test comparing training and test cohorts.

### Training Cohort: NSCLC LOT Algorithm Refinement

#### Overview

The ICCs on the number of LOTs between the LOT algorithm and chart review in the training cohort were 0.81 overall and 0.71 in the complex cases. The prerefinement agreement between the LOT algorithm output and chart review was 91% for the simple cases overall and 61% for the complex cases in the training cohort (Table 3).

**Table 3:** Intraclass correlation coefficients (ICCs) on number of lines of therapy (LOTs) and percentage agreement of non–small cell lung cancer LOT algorithm output with manual chart review.^a^

Group			Training cohort (n=350)					Test cohort (n=150)
			Prerefinement		Postrefinement			
Overall,^b^ ICC^c^ (95% CI)			0.81 (0.77-0.84)		0.87 (0.84-0.89)		0.90 (0.86-0.92)	
Complex cases, ICC (95% CI)			0.71 (0.63-0.78)		0.75 (0.68-0.81)		0.82 (0.73-0.88)	
**Number of LOTs,^d^ percentage agreement (95% CI)**								
	1 (train n=193, test n=87)	88.6 (84.1-93.1)		93.3 (89.7-96.8)		93.1 (87.8-98.4)		
	2 (train n=103, test n=41)	68.0 (58.9-77.0)		72.8 (64.2-81.4)		56.1 (40.9-71.3)		
	3 (train n=36, test n=15)	58.3 (42.2-74.4)		58.3 (42.2-74.4)		53.3 (28.1-78.6)		
	4 (train n=17, test n=3)	23.5 (3.4-43.7)		23.5 (3.4-43.7)		33.3 (0.0-86.7)		
	5 (train n=0, test n=3)	—^e^		—		—		
	6 (train n=1, test n=1)	—		—		—		
Simple cases, overall			91.0 (86.8-95.2)		95.5 (92.5-98.5)		100	
Complex cases, overall			60.5 (53.2-67.8)		64.0 (56.8-71.1)		50.0 (38.6-61.4)	

^a^Simple or complex designation and mutually exclusive groups based on the total number of lines of therapy according to the chart review.

^b^The overall intraclass coefficients included data for simple cases, whereas simple cases were not evaluated separately because of low variability.

^c^ICC: intraclass coefficient.

^d^LOT: line of therapy.

^e^Patient numbers for five and six LOTs were too few for analysis.

For the simple cases, we found that the majority of discordances reflected the LOT not being advanced by chart review but being advanced in the algorithm output because of the 120-day gap-in-therapy rule (Table 4). A minor source of discordance was a difference in the LOT name, specifically when an initial drug was administered but then quickly dropped. Another minor source of discordance involved the 28-day line regimen defining window, that is, when a drug included in an LOT by chart review was not captured in the algorithm output because it had just missed the 28-day window.

**Table 4:** Reasons for discordance between the non–small cell lung cancer line-of-therapy algorithm and blinded chart review: numbers of cases.^a^

Reason for discordance			Training cohort (n=350)										Test cohort (n=150)			
			Prerefinement					Postrefinement					n (%)			N
			n (%)		N		n (%)			N						
**Simple cases, total discordance**			16 (9.0)		178		8 (4.5)			178		0 (0)			76	
	Gap-in-therapy window length	9 (56)		16		3 (38)			8							
	28-day line regimen window	3 (19)		16		3 (38)			8							
	Line name disagreement	3 (19)		16		1 (13)			8							
	Other	1 (6)		6		1 (13)			8							
**Complex cases, total discordance**			68 (39.5)		172		62 (36)			172		37 (50.0)			76	
	Dropped drugs	22 (32)		68		24 (39)			62		17 (46)			37		
	Maintenance therapy classification	14 (21)		68		13 (21)			62		8 (22)			37		
	28-day line regimen window	12 (18)		68		12 (19)			62		6 (16)			37		
	Gap-in-therapy window length	9 (13)		68		4 (6)			62		2 (5)			37		
	Other	11 (16)		68		9 (15)			62		4 (11)			37		

^a^Percentages may not add up to 100 because of rounding.

For the complex cases, the most common source of discordance resulted from dropped drugs, specifically cases when chart review advanced the LOT after a drug in combination therapy was dropped, but the algorithm did not (Table 4). For example, combination therapy with pembrolizumab-carboplatin followed by dropping carboplatin would trigger a new LOT of pembrolizumab monotherapy by chart review but not in the algorithm output.

The second most common source of discordance occurred because of differences in the identification of maintenance therapy, such as the determination of maintenance therapy after L1 regimens by chart review but not in algorithm output. Chart review often labeled maintenance therapy beyond L1 and/or with drugs outside the National Comprehensive Cancer Network (NCCN) guidelines, whereas the algorithm identified maintenance therapy in the L1 setting and using a drug list defined by NCCN guidelines [20,21]. Another reason for maintenance therapy discordance was whether the introduction of a new drug constituted a switch maintenance therapy or a new LOT. We did not attempt to resolve these discordances because of the subjective nature of any decision defining which method produced the true definition of maintenance therapy.

Discordances related to the 120-day gap-in-therapy window and the 28-day regimen window were also relatively common among the complex cases (Table 4).

#### Refinements Made to the NSCLC LOT Algorithm and Results of Refinement

After reviewing the discordances between chart review findings and LOT algorithm output for the training cohort, we used descriptive statistics and plots to determine how to adjust the discordant parameters and improve concordance when possible. For example, we identified the need to increase the gap-in-therapy window from 120 to 180 days by plotting the gap between successive prescriptions, excluding several protein kinase inhibitors as exceptions to the rule for gap-in-therapy line advancement (these *-tinib* drugs target tumor mutations such as *EGFR* and *ALK* genomic aberrations). In addition, we added gemcitabine as a continuation maintenance therapy, implemented the ability to advance the line if a drug in combination therapy were dropped, and implemented the ability to ignore drugs that were dropped during the 28-day line regimen-defining window (Table 5).

**Table 5:** Line-of-therapy algorithm parameters for metastatic non–small cell lung cancer: prerefinement and postrefinement.

Basic modules			Parameters	
			Prerefinement	Postrefinement
L1^a^ first drug			On or after index date^b^	On or after index date^b^
Line regimen window			≤28 days after first drug	≤28 days after first drug
New drug line advancement			First instance	First instance
Exceptions (allowed substitutions)			Cisplatin ↔ carboplatin or paclitaxel ↔ albumin-bound paclitaxel substitution	Cisplatin ↔ carboplatin or paclitaxel ↔ albumin-bound paclitaxel substitution
Gap in therapy window			>120 days	>180 days
Exceptions (allowed gaps)			None	Erlotinib, afatinib, brigatinib, crizotinib, ceritinib, alectinib, gefitinib, osimertinib
**Additional modules**				
	**Maintenance therapy drugs**			
		Continuation maintenance	Bevacizumab, pemetrexed, atezolizumab	Bevacizumab, pemetrexed, atezolizumab, gemcitabine
		Switch maintenance	Pemetrexed, docetaxel	Pemetrexed, docetaxel
	Combination dropped drugs to advance LOT^c^		N/A^d^	Optional flag (not implemented)^e^
	Drug switch during initial regimen window		N/A	Optional flag (not implemented)

^a^L1: first line of therapy.

^b^Index date defined as date of recorded metastatic non–small cell lung cancer diagnosis.

^c^LOT: line of therapy.

^d^N/A: not applicable.

^e^Option included in LOT to handle these cases but not used in this study.

**Figure 3:**
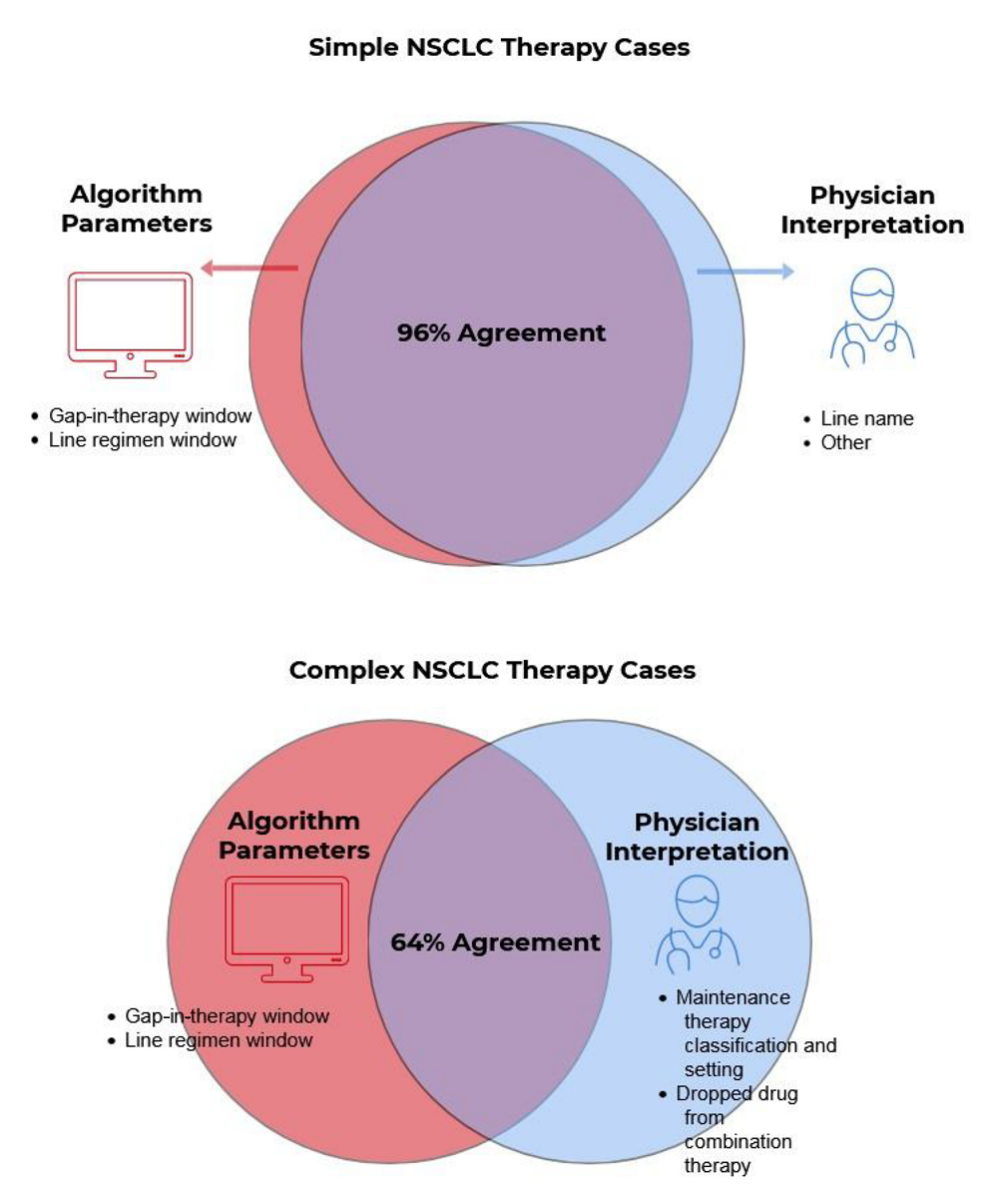
Results of applying the line-of-therapy algorithm to the training cohort postrefinement. NSCLC: non–small cell lung cancer.

Postrefinement agreement increased from 91% to 96% for the simple cases overall and from 61% to 64% for the complex cases, although improvements were limited to receipt of one or two LOTs (Table 3; Figure 3). In addition, postrefinement ICCs increased from 0.81 to 0.87 overall and 0.71 to 0.75 in the complex cases after refinement.

After the LOT algorithm was refined, the total number of discordant results was halved for the simple cases in the training cohort, with the greatest decrease in discordance resulting from the increase from 120 to 180 days in the gap-in-therapy window (Table 4). For the complex cases in the training cohort, discordant numbers decreased only from 68 prerefinement to 62 postrefinement, with most of the decrease resulting from the change in the gap-in-therapy window. However, the number of cases with dropped drugs increased, indicating that fixing one issue can create other issues.

### Test Cohort: Results for the NSCLC LOT Algorithm

The LOT algorithm was then run for the test cohort. For the simple cases, the agreement between the chart review results and algorithm output was 100% (Table 3), with no discordance (Table 4). For the complex cases, agreement was 50% overall and there were 37 instances of discordance, most commonly because of differences resulting from dropped drugs, a pattern similar to that seen for the training cohort. The ICCs were 0.90 overall and 0.82 in the complex cases.

For patients who received one LOT, agreement was high and improved slightly with algorithm refinement (89% prerefinement, 93% postrefinement, 93% test cohort; Table 3). We observed a large decrease in agreement for patients who received more than one LOT.

## Discussion

### Principal Findings

We found an overall good alignment between our automated method of LOT classification and blinded manual chart review. As expected, the likelihood of precise matching between LOT algorithm output and chart review regarding LOT and maintenance therapy identification decreased with an increasing number of unique SACT regimens. This finding is consistent with the simple compounding of errors, that is, the chance of at least one error being found in multiple LOTs is greater than finding an error in a single LOT. On a per-LOT basis, the error would presumably remain fairly constant.

For the purposes of our comparisons, we used manual chart review as the gold standard. We improved the concordance between the LOT algorithm and chart review by increasing the gap-in-therapy window from 120 to 180 days. Concordance was also improved by adding drug class exceptions for protein kinase inhibitors to the gap-in-therapy rule and by adding gemcitabine as a continuation maintenance candidate. Our study notably contributes to the literature insofar as it identifies common sources of discordance between an LOT algorithm and clinician documentation, providing for the possibility of targeted algorithm refinement.

### Addressing Areas of Discordance

Our study is one of the first to validate and refine an open-source LOT algorithm using manual chart review [22]. We note that although there were other potential opportunities to improve the percentage agreement between the algorithm and chart review, we could not identify clear recommendations in the medical literature or among experts to support modifications. Three areas of discordance with the potential to improve agreement included the following: (1) the decision whether to advance the LOT if a drug in a combination regimen is dropped, (2) whether a maintenance therapy could be classified as such beyond the first-line setting, and (3) whether a new drug administration during an LOT constitutes a line advancement or switch maintenance therapy. Although we treated manual chart review as the gold standard, clinical notes do not always document SACT administration in strict accordance with the definitions of LOT and maintenance therapy. Moreover, clinicians may disagree on classifications, leaving room for interpretation.

In the case of (1) whether to advance the line if a drug in a combination regimen is dropped, particular drugs may be dropped because of adverse events. Whether the remaining drugs should be considered the original or a new SACT regimen (LOT) is a subjective matter and may not be explicitly recorded by the prescribing physician. In the case of issues (2) and (3), NCCN guidelines specify that maintenance therapy is prescribed in the first-line setting and that a prescribed set of drugs is eligible for switch maintenance therapy for NSCLC [20,21]. However, these guidelines are not always followed, and maintenance drugs can be prescribed in an atypical manner. Moreover, maintenance therapy is often not recorded as such in clinical notes.

These small apparent inconsistencies may reflect a lack of precise definitions in LOT classification rules, or perhaps more likely, that physicians are instead appropriately focused on dynamically selecting optimal SACT regimens for their patients rather than precisely categorizing LOT and maintenance therapy. In addition, as shown in Tables 3 and 4 by complex cases not improving as the LOT numbers increased, refinements to the algorithm can create other inconsistencies when looking at the entire record. For example, after postrefinement, the algorithm agreed with chart review after exempting *-tinib* drugs from the 180-day rule but added an additional disagreement that resulted from changing the discontinuation gap from 120 to 180 days. Therefore, inconsistencies in physician LOT and maintenance classification, as well as algorithmic edge cases, make it unlikely that an automated LOT algorithm will achieve 100% alignment with independent chart review. In recognition of these unavoidable inconsistencies in LOT classification, we leave many parts of the algorithm to be highly configurable based on the specific use cases of researchers. We anticipate that other groups will make other choices with respect to configuration settings, but our study helps clarify the relative importance of these configuration settings.

Our algorithm is adaptable for use with other cancers and other cancer stages because of its modular design [6]. For example, drug lists, treatment sequences, and temporal parameters, such as the length of the gap between treatments, can be adjusted as appropriate for other tumor types and stages.

### Study Limitations

This study has some limitations. First, we did not consider the length of oral drug administration. Oral drugs are often prescribed with a preset supply, and because the last dose administration is not typically recorded, extrapolation would be needed to determine the length of administration. In this study, our agreement metrics accounted for only the LOT number and regimen, and not the LOT duration; therefore, we considered only the first dose of oral drugs. We note also that we purposely oversampled for complex cases; therefore, the metrics reflect a distribution of patients that was not representative of the overall distribution. For example, only 28% of our selected study population versus 60% of eligible patients in the database received just one LOT without maintenance therapy, our definition of a simple case. Therefore, it is possible that single LOT metrics are under-represented. Finally, it could have been helpful to have more than one physician conducting the manual chart reviews, with an additional independent physician to resolve any discrepancies or disagreements.

Further research is needed on other data sets to determine if the results and conclusions are generalizable. In addition, considerations such as detecting drug cycles and accounting for drug-specific nuances may increase the robustness of the algorithm. Further research on the appropriate metrics and benchmarks may be needed to address issues such as error compounding.

### Conclusions

This study validates an EHR- and claims-based algorithm using medical chart review. We have refined the algorithm, highlighted areas of discordance, and noted the error compounding on further lines, allowing a deeper understanding of how the LOT algorithm may be used. We envision contributions to different disease indications and areas. In addition, common data set benchmarks, metrics, and increased accessibility will contribute substantially toward the development and adoption of this tool. Finally, a database of specific business rules concerning individual drugs and other nuanced behaviors will increase the robustness of the algorithm.

## Appendix

Multimedia Appendix 1 Identification of patients with metastatic non–small cell lung cancer.

## Abbreviations

ALK: anaplastic lymphoma kinase
ANOVA: analysis of variance
EGFR: epidermal growth factor receptor
EHR: electronic health record
L1, L2, L3, L4: first-, second-, third-, and fourth-line therapy
LOT: line of therapy
NCCN: National Comprehensive Cancer Network
NSCLC: non–small cell lung cancer
SACT: systemic anticancer therapy
