# Supplementary material for: An Automated Line-of-Therapy Algorithm for Adults With Metastatic Non–Small Cell Lung Cancer: Validation Study Using Blinded Manual Chart Review
Source: JMIR Med Inform. 2021 Oct 12;9(10):e29017. doi: 10.2196/29017 (PMC8548977; doi:10.2196/29017)
Supplement: Multimedia Appendix 1 [file medinform_v9i10e29017_app1.docx]

## Appendix A. Identification of patients with metastatic NSCLC

For the purpose of validating the LOT algorithm, we identified adult patients treated for metastatic NSCLC with SACT. As a first step, we extracted adult patient records with evidence of a malignant neoplasm of trachea, bronchus, and/or lung, defined by two or more diagnosis codes (ICD-9 code 162.x or ICD-10 codes C34.x, C39.9) separated in time by ≥30 days in the 7.5-year period from January 1, 2011, to June 30, 2018. We limited eligibility to individuals with at least one clinical document referencing NSCLC (ie, at least one document that included one of the following text strings: "non small" or "non-small'' or "NSCLCA" or "NSCLC"). We further limited inclusion to patients with (1) evidence of metastatic disease per ICD codes and/or text search and (2) SACT orders occurring on or after the first metastatic diagnosis. We excluded patients who had received any SACT commonly used for small cell lung cancer: namely, platinum + etoposide, platinum + irinotecan, topotecan, cyclophosphamide + doxorubicin + vincristine + temozolomide, ifosfamide, or bendamustine.
